# Integrated Genetics and Micronutrient Data to Inform the Causal Association Between Serum Calcium Levels and Ischemic Stroke

**DOI:** 10.3389/fcell.2020.590903

**Published:** 2020-11-11

**Authors:** Qiang Meng, Lu Huang, Kai Tao, Yong Liu, Jiangpeng Jing, Wen Wang, Huaizhou Qin, Dayun Feng, Qing Cai

**Affiliations:** ^1^Department of Neurosurgery and Institute for Functional Brain Disorders, Tangdu Hospital, Fourth Military Medical University, Xi’an, China; ^2^Department of Neurosurgery, The First Affiliated Hospital of Xi’an Jiaotong University, Xi’an, China; ^3^Department of Radiology and Functional and Molecular Imaging Key Lab of Shaanxi Province, Tangdu Hospital, Fourth Military Medical University, Xi’an, China

**Keywords:** serum calcium, ischaemic stroke, Mendelian randomization, genome-wide association study, coronary artery disease

## Abstract

There has been an increased interest for observational studies or randomized controlled trials exploring the impact of calcium intake on cardiovascular diseases (CVD) including coronary artery disease (CAD) and ischemic stroke (IS). However, a direct relationship between total calcium intake and CVD has not been well established and remains controversial. Mendelian randomization (MR) studies have been performed to evaluate the causal association between serum calcium levels and CAD risk and found that increased serum calcium levels could increase the risk of CAD. However, MR analysis found no significant association between genetically higher serum calcium levels and IS as well as its subtypes. Hence, three MR studies reported inconsistent effects of serum calcium levels on CAD and IS. Here, we performed an updated MR study to investigate the association of serum calcium levels with the risk of IS using large-scale genome-wide association study (GWAS) datasets. We selected 14 independent genetic variants as the potential instrumental variables from a large-scale serum calcium GWAS dataset and extracted summary statistics corresponding to the 14 serum calcium genetic variants from the MEGASTROKE Consortium IS GWAS dataset. Interestingly, we found a significant association between serum calcium levels and IS risk using the robust inverse-variance weighted (IVW) and penalized robust IVW methods, with β = 0.243 and *P* = 0.002. Importantly, the MR results from the robust MR-Egger and penalized robust MR-Egger methods further supported the causal association between serum calcium levels and IS risk, with β = 0.256 and *P* = 0.005. Meanwhile, the estimates from other MR methods are also consistent with the above findings.

## Introduction

In recent years, there has been an increased interest for observational studies or randomized controlled trials exploring the impact of calcium intake on cardiovascular diseases (CVD) including coronary artery disease (CAD) and ischemic stroke (IS; Heaney et al., 2012; Anderson et al., 2016; Tankeu et al., 2017). In fact, a significant number of studies have reported an association between calcium intake and adverse CVD (Bolland et al., 2008, 2010, 2011; Tankeu et al., 2017). However, these conclusions have been widely questioned by a number of experts who have raised concerns about the methodology, potential biases, and confounders (Lappe and Heaney, 2008; Puccetti, 2008; Ramlackhansingh, 2008; Grove and Cook, 2010; Heiss et al., 2010; Black, 2011). Until now, a direct relationship between total calcium intake and CVD has not been well established and remains controversial, as described in two recent reviews (Heaney et al., 2012; Tankeu et al., 2017).

Until recently, Mendelian randomization (MR) studies have been performed to evaluate the causal association between increased serum calcium levels and CAD risk (Xu et al., 2017; Larsson et al., 2017a). Xu et al. (2017) selected four independent variants for the main analysis and 13 correlated variants for a sensitivity analysis as the included instrumental variables. All these genetic variants could influence the serum calcium levels, with the genome-wide significance (*P* < 5.00E-08) from a recent genome-wide association study (GWAS) including 20,611 individuals of European ancestry (O’Seaghdha et al., 2010). Larsson et al. (2017a) selected seven independent genetic variants influencing serum calcium levels, with the genome-wide significance (*P* < 5.00E-08) from a recent GWAS including 61,079 individuals of European ancestry (O’Seaghdha et al., 2013), as the instrumental variables. Both Xu et al. (2017) and Larsson et al. (2017a) identified that increased serum calcium levels could increase the risk of CAD.

Importantly, MR analysis has also been performed to evaluate the causal association between increased serum calcium levels and IS risk (Larsson et al., 2019). Larsson et al. (2019) selected seven independent genetic variants influencing serum calcium levels and a large-scale IS dataset from the MEGASTROKE Consortium (34,217 cases and 404,630 controls). However, they found no significant association between genetically higher serum calcium levels and IS as well as its subtypes (Larsson et al., 2019). Hence, these three MR studies reported inconsistent effects of serum calcium levels on CAD and IS (Xu et al., 2017; Larsson et al., 2017a, 2019). Here, we performed an updated MR study to investigate the association of serum calcium levels with the risk of stroke using large-scale serum calcium GWAS dataset and IS GWAS dataset.

## Materials and Methods

### Study Design

The MR study design has been well established in recent studies (Cheng et al., 2018; Zhuang et al., 2019a,b; Sun et al., 2020). In brief, we only selected the GWAS summary datasets about serum calcium levels and IS (O’Seaghdha et al., 2013; Malik et al., 2018). Informed consent was provided by all participants in all these corresponding original studies (O’Seaghdha et al., 2013; Malik et al., 2018).

### Serum Calcium Genetic Variants

We selected 14 independent genetic variants as the potential instrumental variables from a large-scale serum calcium GWAS dataset (O’Seaghdha et al., 2013). In brief, this dataset consisted of 61,079 individuals of European descent, including 39,400 individuals in the discovery stage and 21,679 individuals in the replication stage (O’Seaghdha et al., 2013). A linear regression method was used to evaluate the association of each genetic variant with serum calcium level using an additive genetic effect by adjusting some key covariates including age, sex, principal components, and study center (O’Seaghdha et al., 2013). Of these 14 genetic variants, eight were associated with serum calcium levels with *P* < 5.00E-08 and six genetic variants were associated with serum calcium levels with *P* < 1.00E-04 (O’Seaghdha et al., 2013). We provide more detailed information about these 14 variants in Table 1. Recent studies have provided more detailed information about this dataset (O’Seaghdha et al., 2013; Xu et al., 2017; Larsson et al., 2017a, 2019).

**TABLE 1:** Characteristics of the 14 genetic variants associated with serum calcium levels.

SNP	Nearby genes	EA	NEA	EAF	β (mg/dl)	SE	*P* value	*R*^2^ (%)
rs10491003	GATA3	T	C	0.09	0.027	0.005	4.80E-09	0.05
rs11967485	*ARID1B*	g	a	0.9	0.026	0.005	9.40E-07	0.05
rs12150338	*WDR81/SERPINF2*	t	c	0.09	0.03	0.006	1.50E-06	0.06
rs1550532	DGKD	C	G	0.31	0.018	0.003	8.20E-11	0.06
rs1570669	CYP24A1	G	A	0.34	0.018	0.003	9.10E-12	0.06
rs17711722	VKORC1L1	T	C	0.47	0.015	0.003	8.20E-09	0.04
rs1801725	CASR	T	G	0.15	0.071	0.004	8.90E-86	0.51
rs2281558	*PYGB*	t	g	0.25	0.015	0.003	5.10E-06	0.03
rs2885836	*RARBTOP2B*	a	g	0.24	0.012	0.003	5.40E-05	0.02
rs4074995	*RGS14/SLC34A1*	a	g	0.28	0.013	0.003	4.60E-06	0.03
rs7336933	DGKH/KIAA0564	G	A	0.85	0.022	0.004	9.10E-10	0.05
rs7481584	CARS	G	A	0.7	0.018	0.003	1.20E-10	0.05
rs780094	GCKR	T	C	0.42	0.017	0.003	1.30E-10	0.06
rs9447004	*CD109*	a	g	0.48	0.012	0.003	3.30E-06	0.03

*SNP, single nucleotide polymorphism; *EA*, effect allele; *NEA*, non-effect allele; *EAF*, effect allele frequency; *SE*, standard error; β, regression coefficient based on the effect allele; and R^2^, proportion of serum calcium variance explained by the selected 14 genetic variants.*

### IS GWAS Dataset

The IS GWAS dataset is from a large-scale multi-ancestry stroke GWAS of 67,162 cases and 454,450 controls from the MEGASTROKE Consortium (Malik et al., 2018). The participants are of multiple ancestries including European, African, South Asian, mixed Asian, and Latin American (Malik et al., 2018). Before the normal GWAS analysis, the MEGASTROKE Consortium conducted genotyping, imputation, and quality control analyses (Malik et al., 2018). They further conducted a fixed-effects inverse-variance weighted (IVW) meta-analysis using METAL in each ancestral group and then performed a meta-analysis of the ancestry-specific meta-analysis results (Malik et al., 2018). In order to be consistent with the serum calcium GWAS dataset, we limit our follow-up analysis to samples of European ancestry, including 34,217 IS cases and 406,111 controls (Malik et al., 2018). More detailed information about the MEGASTROKE dataset has been widely described in the original study (Malik et al., 2018) and in the recent MR study (Larsson et al., 2019).

### Pleiotropy Analysis

Using the summary results of these 14 genetic variants in both serum calcium levels and IS GWAS datasets, we first conducted a pleiotropy analysis using two statistical methods including the MR-Egger intercept test and the MR pleiotropy residual sum and outlier (MR-PRESSO) test (Verbanck et al., 2018). Both methods have been widely used in recent MR studies (Liu et al., 2018; Larsson et al., 2019; He et al., 2020; Wang et al., 2020). The statistical significance for evidence of pleiotropy is *P* < 0.05.

### MR Analysis

For MR analysis, we selected 11 MR methods including simple median, weighted median, penalized weighted median, IVW, penalized IVW, robust IVW, penalized robust IVW, MR-Egger, penalized MR-Egger, robust MR-Egger, and penalized robust MR-Egger (Larsson et al., 2019; He et al., 2020). IVW is a standard MR analysis method. For multiple independent genetic variants, IVW could weigh the average of these single causal estimates using the inverse of their approximate variances as weights (Burgess and Thompson, 2017; Larsson et al., 2019; He et al., 2020). The causal estimate from the MR-Egger is obtained using the same regression model as the weighted linear regression described above, but allowing the intercept to be estimated as part of the analysis (Burgess and Thompson, 2017). The simple median estimator is calculated as the median of the Wald ratio estimates [ratio of single nucleotide polymorphism (SNP) on outcome to SNP on calcium]. A weighted median of these causal estimates could be considered to account for differences in the precision of estimates and could provide consistent estimates even if 50% of the instrumental variables are invalid (Burgess et al., 2017). The robust option, penalized option, and the penalized option of the weighted median, IVW, and MR-Egger are all improved MR methods (Yavorska and Burgess, 2017).

### Statistical Analysis

The odds ratio (OR) as well as the 95% confidence interval (CI) of IS correspond to per 0.5 mg/dl increase [about 1 standard deviation (SD)] in serum calcium levels. The significance threshold was *P* < 0.05. All statistical tests were completed by R (version 3.5.1) and R package “MendelianRandomization” (Yavorska and Burgess, 2017).

### Power Analysis

The proportion of serum calcium variance explained by the selected 14 genetic variants could be estimated using *R*^2^.

$$R^{2}=\sum_{i=1}^{14}\frac{\beta_{i}^{2}\times 2\times MAF_{SNP_{i}}\left(1-MAF_{SNP_{i}}\right)}{SD^{2}}$$

where β is the effect size (beta coefficient) between *SNP*_i_ and the serum calcium levels, *MAF*_*SNP_i*_ is the minor allele frequency for *SNP*_i_, and SD = 0.5 mg/dl (Larsson et al., 2017a; He et al., 2020). Using *R*^2^ and other necessary information, we performed a power analysis using mRnd: Power calculations for MR^1^ (Brion et al., 2013). The input variables include the sample size, type I error rate, proportion of cases in the study, true odds ratio of the outcome variable per standard deviation of the exposure variable, and the proportion of variance explained for the association between the SNP and the exposure variable (Brion et al., 2013).

## Results

### Association of 14 Serum Calcium Variants With IS

In the IS GWAS dataset from the MEGASTROKE Consortium, we extracted the summary statistics corresponding to the 14 serum calcium genetic variants. The results indicated that none of these 14 variants was significantly associated with IS (*P* < 0.05; Table 2).

**TABLE 2:** Characteristics of the 14 genetic variants associated with IS risk.

SNP	EA	NEA	EAF	β	SE	*P* value
rs11967485	a	g	0.1093	−0.0063	0.0169	0.7097
rs1801725	t	g	0.1379	0.018	0.0151	0.2341
rs2281558	t	g	0.2614	0.0068	0.0113	0.5473
rs12150338	t	c	0.0979	0.0037	0.0186	0.8415
rs4074995	a	g	0.2943	0.0009	0.011	0.9363
rs9447004	a	g	0.4968	−0.0047	0.0109	0.6674
rs17711722	t	c	0.3936	0.0048	0.0129	0.710
rs1570669	a	g	0.6604	0.0191	0.0105	0.06948
rs7481584	a	g	0.2974	−0.0105	0.0112	0.3489
rs7336933	a	g	0.148	−0.0014	0.0135	0.9146
rs10491003	t	c	0.0913	−0.0327	0.0178	0.06655
rs2885836	a	g	0.2278	0.0058	0.0122	0.6333
rs780094	t	c	0.3917	0.0059	0.0111	0.5976
rs1550532	c	g	0.3201	0.0022	0.0106	0.8345

*SNP, single nucleotide polymorphism; *EA*, effect allele; *NEA*, non-effect allele; *EAF*, effect allele frequency; β, regression coefficient based on the effect allele; *SE*, standard error; and *IS*, ischemic stroke.*

### Statistical Analysis

The pleiotropy analysis using the MR-Egger intercept test indicated no significant pleiotropy, with intercept = −0.004 and *P* = 0.574. The pleiotropy analysis using the MR-PRESSO test further indicated no significant pleiotropy, with *P* = 0.669. Hence, all these 14 variants are valid instrumental variables and could be used in the MR analysis.

**FIGURE 1:**
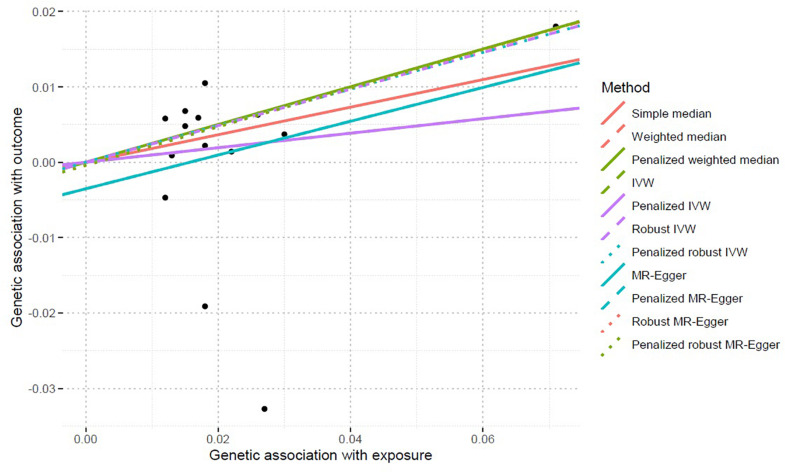
Single MR estimates from each of the 14 genetic variants using 11 MR methods. *MR*, Mendelian randomization; *IVW*, inverse-variance weighted. The *black scatter plots* indicate single causal estimates from each of the 14 genetic variants associated with serum calcium levels on the *x*-axis and IS risk on the *y*-axis. The *continuous line* represents the causal effect of serum calcium levels on IS risk.

Inverse-variance weighted indicated no significant association between serum calcium levels and IS risk, with β = 0.096 and *P* = 0.499. Interestingly, we found a significant association between serum calcium levels and IS risk using the robust IVW and penalized robust IVW methods, with β = 0.243 and *P* = 0.002 (Table 3). Importantly, the MR results from the robust MR-Egger and penalized robust MR-Egger methods further supported the causal association between serum calcium levels and IS risk, with β = 0.256 and *P* = 0.005 (Table 3). Meanwhile, the estimates from the other MR methods are also consistent with the above findings, as provided in Table 3. We also provided the single MR estimates from each of the 14 genetic variants using 11 MR methods, as described in Figure 1.

**TABLE 3:** MR analysis results using different methods.

			95% CI	95% CI	
MR method	β	SE	(Down)	(Up)	*P* value
Simple median	0.183	0.223	–0.254	0.62	0.412
Weighted median	0.249	0.19	–0.124	0.622	0.19
Penalized weighted median	0.25	0.191	–0.123	0.624	0.189
IVW	0.096	0.142	–0.182	0.375	0.499
Penalized IVW	0.096	0.142	–0.182	0.375	0.499
Robust IVW	0.243	0.078	0.089	0.396	0.002
Penalized robust IVW	0.243	0.078	0.089	0.396	0.002
MR-Egger	0.224	0.268	–0.302	0.75	0.404
Penalized MR-Egger	0.224	0.268	–0.302	0.75	0.404
Robust MR-Egger	0.256	0.091	0.076	0.435	0.005
Penalized robust MR-Egger	0.256	0.091	0.076	0.435	0.005

*MR, Mendelian randomization; *IVW*, inverse-variance weighted; β, regression coefficient; *SE*, standard error; and *CI*, confidence interval. Significance level 0.05.*

### Statistical Power

These 14 genetic variants could explain 1.10% of the serum calcium variance (*R*^2^ = 1.10%). Power analysis using mRnd indicated that our MR study had 80% power to detect effect sizes of moderate magnitude, with ORs as low as 0.83 and as high as 1.15 per SD (0.5 mg/dl) increase in serum calcium levels for IS. The power is 100% to detect the causal association, with OR = 1.28 (β = 0.243).

## Discussion

Until now, meta-analyses of randomized controlled trials have not demonstrated convincing evidence that calcium intake (diet and supplements) could improve stroke (Heaney et al., 2012; Tankeu et al., 2017). Some studies even reported an association between calcium intake and adverse stroke outcomes (Bolland et al., 2008, 2010, 2011). In 2018, Jenkins et al. (2018) performed a meta-analysis of individual randomized controlled trials from previous meta-analyses and additional searches. They found that calcium supplements showed no consistent benefit for the prevention of stroke, nor was there a benefit for all-cause mortality to support their continued use (Jenkins et al., 2018).

Until recently, MR studies have been performed to evaluate the causal effects of high serum calcium levels on the risk of CAD and IS. However, these MR studies reported inconsistent findings. Some studies indicated that high serum calcium levels contributed to an increased risk of CAD (Xu et al., 2017; Larsson et al., 2017a). One MR study indicated no significant association between high serum calcium levels and IS as well as its subtypes (Larsson et al., 2019). Hence, these inconsistent findings drove us to conduct an updated MR analysis, and we found a significant association between high serum calcium levels and increased IS risk.

There are two main differences between our MR study and previous MR studies (Xu et al., 2017; Larsson et al., 2017a, 2019). Firstly, we selected 14 genetic variants associated with serum calcium levels as the potential instrumental variables, as these 14 genetic variants could explain 1.10% of the serum calcium variance. In 2017, Larsson et al. (2017a) selected seven genetic variants and excluded the rs780094 variant to evaluate the causal association between serum calcium levels and CAD, as it had a pleiotropic association with cardiometabolic risk factors. The remaining six genetic variants only explained about 0.8% of the variation in serum calcium levels (Larsson et al., 2017a). In 2019, Larsson et al. (2019) selected seven genetic variants to evaluate the causal association between serum calcium levels and IS risk, which could explain 0.9% of the variance in serum calcium levels. In 2017, Xu et al. (2017) only selected four variants influencing serum calcium levels as the instrumental variables. Hence, our MR study has the largest explained variation in serum calcium levels, which may further contribute to identify positive findings.

Secondly, we selected more MR methods than did the previous MR studies (Xu et al., 2017; Larsson et al., 2017a, 2019). In our MR study, we selected a total of 11 MR methods including simple median, weighted median, penalized weighted median, IVW, penalized IVW, robust IVW, penalized robust IVW, MR-Egger, penalized MR-Egger, robust MR-Egger, and penalized robust MR-Egger. Importantly, all these MR estimates were consistent with each other in terms of direction and magnitude, as provided in Table 3. In 2017, Larsson et al. (2017a) selected three MR methods including IVW, weighted median, and MR-Egger. In 2017, Xu et al. (2017) selected four MR methods including weighted generalized linear regression, IVW, weighted median, and MR-Egger regression. In 2019, Larsson et al. (2019) selected five MR methods including IVW, weighted median, heterogeneity-penalized model averaging method, MR-Egger, and MR-PRESSO.

Hence, our findings are consistent with recent findings from MR analysis in CAD (Xu et al., 2017; Larsson et al., 2017a). Meanwhile, our findings are consistent with previous observational studies (Chung et al., 2015). Chung et al. measured the levels of serum calcium and albumin-corrected calcium in 1,915 participants (Chung et al., 2015). They found that the serum calcium level was significantly associated with increased modified Rankin scale (MRS) [1.19 (1.03–1.38)]. The albumin-corrected calcium level was also significantly associated with increased MRS [1.21 (1.01–1.44)] (Chung et al., 2015). The authors further identified that a high albumin-corrected serum calcium level could cause a poorer short-term outcome and increase the long-term mortality in acute IS (Chung et al., 2015). In 2017, de Abajo et al. (2017) performed a nested case–control study to evaluate the risk of IS with calcium supplement using 2,690 IS cases and 19,538 controls. Their results showed that calcium supplement was associated with an increased trend of IS risk in the whole population (OR = 1.18, 95% CI = 0.86–1.61, and *P* = 0.31).

Our MR study also has some limitations. One limitation is that not all of the selected 14 genetic variants are associated with serum calcium levels with genome-wide significance threshold *P* < 5.00E-08. Only eight genetic variants were associated with serum calcium levels with *P* < 5.00E-08 and six genetic variants were associated with serum calcium levels with *P* < 1.00E-04 (O’Seaghdha et al., 2013). The other limitation is that we only selected two statistical methods—the MR-Egger intercept test and the MR-PRESSO test—to conduct a pleiotropy analysis and test the confounders. Interestingly, we did not find any significant pleiotropy using both methods. However, the statistical methods could not completely exclude all confounders. It is a general challenge in current MR studies (Emdin et al., 2017; Larsson et al., 2017a, 2019; He et al., 2020). Hence, follow-up studies are necessary to replicate our findings.

In summary, our MR study provides genetic evidence that high serum calcium levels are significantly associated with an increased risk of IS in the general population.

## Data Availability Statement

All datasets presented in this study are included in the article/supplementary material.

## Author Contributions

QC and DF designed the project. QM, LH, and KT analyzed the data. All authors wrote and approved the manuscript.

## Conflict of Interest

The authors declare that the research was conducted in the absence of any commercial or financial relationships that could be construed as a potential conflict of interest.

## Footnotes

1 https://shiny.cnsgenomics.com/mRnd/
