# Corrigendum: Integrated Genetics and Micronutrient Data to Inform the Causal Association Between Serum Calcium Levels and Ischemic Stroke

**DOI:** 10.3389/fcell.2020.634957

**Published:** 2020-12-18

**Authors:** Qiang Meng, Lu Huang, Kai Tao, Yong Liu, Jiangpeng Jing, Wen Wang, Huaizhou Qin, Dayun Feng, Qing Cai

**Affiliations:** ^1^Department of Neurosurgery and Institute for Functional Brain Disorders, Tangdu Hospital, Fourth Military Medical University, Xi'an, China; ^2^Department of Neurosurgery, The First Affiliated Hospital of Xi'an Jiaotong University, Xi'an, China; ^3^Department of Radiology and Functional and Molecular Imaging Key Lab of Shaanxi Province, Tangdu Hospital, Fourth Military Medical University, Xi'an, China

**Keywords:** serum calcium, ischaemic stroke, Mendelian randomization, genome-wide association study, coronary artery disease

In the published article, there was an error regarding the affiliation(s) for Lu Huang, Kai Tao, Huaizhou Qin, Dayun Feng, Qing Cai. Instead of 2, it should be 1. Meanwhile, there was an error regarding the affiliation(s) for Yong Liu, Jiangpeng Jing. Instead of 1, it should be 2.

In the published article, there was an error regarding the affiliation(s) for Qiang Meng. As well as having affiliation(s) 1, they should also have 2.

The authors apologize for this error and state that this does not change the scientific conclusions of the article in any way. The original article has been updated.

